# TREM2-LRP1 axis in Alzheimer's disease: pharmacological trade-offs of receptor shedding and functional restoration

**DOI:** 10.1007/s00018-026-06307-w

**Published:** 2026-08-07

**Authors:** Yuji Kamikubo

**Affiliations:** Department of Pharmacology, Juntendo University Faculty of Medicine, 2-1-1, Hongo Bunkyo-Ku, Tokyo, 113-8421 Japan

**Keywords:** Blood–brain barrier, ADAM10/17, Cholesterol esterification, sTREM2, Lipid droplets, Neuroinflammation

## Abstract

The interplay between triggering receptor expressed on myeloid cells 2 (TREM2) and low-density lipoprotein receptor-related protein 1 (LRP1) is critical for amyloid-beta (Aβ) clearance in Alzheimer’s disease (AD). Current therapeutic strategies targeting this axis can be broadly divided into two pharmacological approaches: indirect induction via metabolic modulation (e.g., ACAT1 inhibition) and direct functional restoration of LRP1. While ACAT1 inhibition effectively upregulates LRP1, recent findings suggest that this effect is mechanistically coupled to ADAM10/17-mediated shedding of TREM2. This raises a critical pharmacological dilemma: balancing the benefit of LRP1 upregulation against the reduction of cell-surface TREM2. While TREM2 shedding may represent a functional shift from membrane-bound sensing to soluble Aβ clearance rather than a simple loss of function, cell-surface TREM2 signaling remains essential for Aβ sensing and microglial fitness in the long term. Conversely, strategies focusing on the direct restoration of LRP1 transport achieve robust Aβ clearance but face challenges regarding delivery specificity. Integrating emerging evidence on lipid droplet–driven microglial dysfunction and membrane fluidity, this article discusses the trade-offs of these strategies and proposes that preserving surface TREM2 signaling while enhancing LRP1 activity is key to long-term therapeutic efficacy. Clinical evidence from the INVOKE-2 trial further supports the need for combination strategies targeting both TREM2 signaling and LRP1-mediated clearance.

## Introduction: the intersection of lipid metabolism and innate immunity

While anti-amyloid antibodies are now clinically approved, the search for safer and more sustainable disease-modifying therapies for Alzheimer’s disease (AD) remains pressing. Attention has increasingly shifted toward enhancing endogenous clearance mechanisms in the brain, particularly those governed by microglia and the blood–brain barrier (BBB). Recent multi-omics studies have converged on microglial lipid dysregulation as a key driver of pathology. Xu et al. [1] demonstrated that microglia-specific lipid accumulation accelerates disease progression, and Prakash et al. [2] revealed that lipid droplet (LD) accumulation mediated by diacylglycerol O-acyltransferase 2 (DGAT2) directly impairs phagocytic capacity, thereby compromising triggering receptor expressed on myeloid cells 2 (TREM2)-mediated amyloid-beta (Aβ) sensing [3, 4] and the endogenous clearance capacity linked to low-density lipoprotein receptor-related protein 1 (LRP1)-dependent Aβ efflux at the BBB [5, 6]. Within this context, the axis formed by TREM2 and LRP1 emerges as a central pharmacological target. TREM2 functions as a sensor for pathology-associated lipids and Aβ [3, 4], whereas LRP1 orchestrates major clearance transport at the BBB [5, 6]. For drug development, the unresolved question is not whether this axis should be targeted, but how—through metabolic stress–induced adaptation or through direct functional restoration.

## The indirect approach: ACAT1 inhibition and the “shedding” mechanism

Modulation of cholesterol esterification has emerged as a promising therapeutic strategy. Inhibitors of acyl-coenzyme A: cholesterol acyltransferase 1 (ACAT1; also known as SOAT1), such as avasimibe, reduce cholesteryl ester levels, suppress neuroinflammation, and enhance autophagic flux [7, 8]. Recently, Hovde et al. [9] identified an unexpected mechanistic link between ACAT1 inhibition and Aβ clearance. They showed that ACAT1 blockade promotes shedding of the TREM2 ectodomain mediated by a disintegrin and metalloproteinase 10 and 17 (ADAM10/17), generating soluble TREM2 (sTREM2). This released sTREM2 forms complexes with Aβ, facilitating Aβ uptake via upregulated LRP1. Mechanistically, this process is likely driven by alterations in membrane lipid composition and fluidity. As demonstrated by Kojro et al. [10] in the context of APP cleavage, reduced membrane cholesterol enhances ADAM10 activity, thereby promoting cleavage of surface substrates. Although this represents a mechanistic analogy, the same ADAM10-mediated shedding of TREM2 following ACAT1 inhibition has been directly demonstrated [9]. While this mechanism provides a coherent explanation for the efficacy of avasimibe, it introduces a critical functional trade-off. The pathway is inherently dependent on proteolytic loss of surface TREM2, resulting in a substantial reduction in receptor density (approximately 47% in mouse BV2 microglial cells treated with avasimibe at 10 µM [9]). Rather than representing a simple loss of function, this shedding may reflect a functional shift from membrane-bound sensing to soluble Aβ clearance [11, 12]. However, such a shift comes at a cost, as the sustained reduction of surface TREM2 compromises membrane-bound signaling competency. Zhao et al. [3] demonstrated that direct interaction between Aβ and surface TREM2 is required to initiate intracellular signaling cascades (e.g., SYK/GSK3β) that support microglial activation, survival, and migration to amyloid deposits. Thus, a strategy that chronically relies on TREM2 shedding risks undermining microglial sensing capacity, potentially promoting a progressive loss-of-function phenotype.

## The direct approach: functional restoration of LRP1

Conversely, direct targeting of LRP1 offers a mechanistically distinct strategy. Deane et al. [6] established that LRP1 mediates Aβ efflux across the BBB, a function that declines with aging and AD progression [5]. More recently, Chen et al. [13] demonstrated that LRP1 function can be restored using LRP1-targeted polymersomes. By biasing LRP1 trafficking toward transcytosis, this approach achieved rapid systemic Aβ clearance and concomitant cognitive improvement. Importantly, this strategy enhances transporter efficiency without necessitating receptor shedding or metabolic stress responses. Nevertheless, direct modulation of LRP1 is not without limitations. LRP1 plays essential roles in multiple physiological processes beyond Aβ clearance, raising concerns about off-target effects. In addition, the clinical translation of nanocarrier-based systems faces practical challenges inherent to nanomedicine development, when compared with conventional small-molecule inhibitors.

## Discussion: navigating the pharmacological trade-offs

**Fig. 1:**
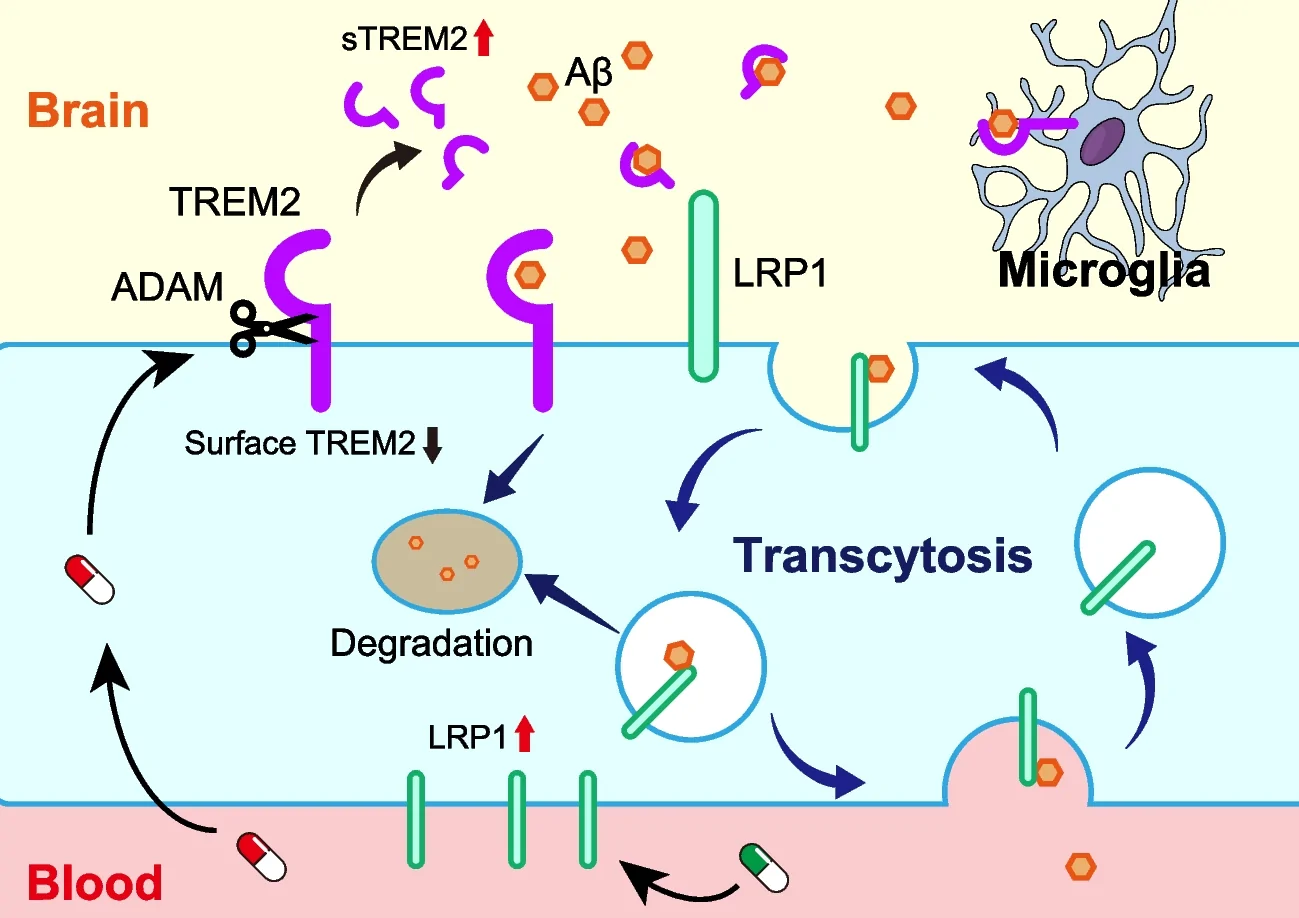
Schematic representation of the pharmacological trade-offs in the TREM2-LRP1 axis. The figure illustrates two distinct therapeutic strategies across the blood–brain barrier (Blood/Brain interface). The indirect approach (e.g., ACAT1 inhibition, depicted as red/white capsules) triggers ADAM-mediated cleavage of membrane-bound TREM2. While this generates sTREM2, which may bind Aβ, it leads to a critical reduction in surface TREM2 density (indicated by the downward arrow), compromising microglial sensing. The direct approach targeting LRP1 (depicted as green/white carriers) promotes LRP1-mediated transcytosis and intracellular degradation of Aβ. This strategy enhances clearance efficiency while preserving cell-surface TREM2 integrity

Collectively, these findings point to a fundamental pharmacological choice: inducing TREM2 shedding to trigger LRP1-mediated uptake (indirect strategy) versus restoring LRP1 transporter function directly (direct strategy) (Fig. 1). ACAT1 inhibition effectively alleviates intracellular lipid overload, releasing microglia from a senescent-like metabolic state. However, if this benefit is mechanistically coupled to sustained TREM2 shedding [9], long-term depletion of surface TREM2 may compromise microglial fitness and responsiveness. We argue that the optimal therapeutic strategy lies in functionally uncoupling lipid normalization from receptor shedding. Specifically, lipid droplet resolution should be achieved without excessive perturbation of membrane cholesterol that would activate ADAM10/17. Potential approaches include:

- Targeting downstream lipid regulators: Inhibition of enzymes such as DGAT2 [2] may resolve lipid droplets without significantly altering membrane cholesterol pools.
- Dosing precision: Careful titration of ACAT1 inhibitors to remain below the threshold that triggers extensive surface cleavage (i.e., the ~ 47% reduction in membrane TREM2 observed with avasimibe at 10 µM [9]), as chronic loss of surface TREM2 risks undermining microglial signaling and Aβ clearance capacity [3, 4].
- Combination strategies: Pairing metabolic modulators with LRP1-targeting approaches [13] or TREM2 agonistic antibodies [14] to independently enhance clearance and signaling, thereby avoiding a pharmacological “zero-sum game.” Notably, the failure of the TREM2-targeting antibody AL002 to meet primary endpoints in the INVOKE-2 trial [12, 14] suggests that preserving or activating surface TREM2 alone may be insufficient, and that combination strategies targeting both TREM2 signaling and LRP1-mediated clearance may be essential for sustained therapeutic efficacy.

## Concluding remarks

The identification of the ACAT1–sTREM2–LRP1 axis provides important insights into microglial immunometabolism. However, therapeutic strategies that trade cell-surface sensors (TREM2) for short-term gains in clearance must be carefully weighed against approaches that directly restore transporter function (LRP1). Preserving surface TREM2 signaling while harnessing the benefits of lipid regulation is likely to be essential for durable efficacy in AD drug development. Nevertheless, as underscored by the disappointing outcomes of the INVOKE-2 trial [12, 14], preserving surface TREM2 signaling alone is unlikely to be sufficient; rather, it should be regarded as one component of a broader combination strategy that also targets LRP1-mediated clearance and lipid homeostasis.

## Data Availability

Not applicable. This article is a theoretical perspective and does not contain any original research data.
